# Neuroanatomical differences in visual, motor, and language cortices between congenitally deaf signers, hearing signers, and hearing non-signers

**DOI:** 10.3389/fnana.2013.00026

**Published:** 2013-08-02

**Authors:** John S. Allen, Karen Emmorey, Joel Bruss, Hanna Damasio

**Affiliations:** ^1^Dornsife Cognitive Neuroscience Imaging Center, University of Southern CaliforniaLos Angeles, CA, USA; ^2^Brain and Creativity Institute, University of Southern CaliforniaLos Angeles, CA, USA; ^3^Laboratory for Language and Cognitive Neuroscience, San Diego State UniversitySan Diego, CA, USA; ^4^Laboratory of Computational Neuroimaging, Department of Neurology, University of Iowa Carver College of MedicineIowa City, IA, USA

**Keywords:** deaf, sign language, inferior frontal gyrus, motor hand region, calcarine, morphometry

## Abstract

We investigated effects of sign language use and auditory deprivation from birth on the volumes of three cortical regions of the human brain: the visual cortex surrounding the calcarine sulcus in the occipital lobe; the language-related cortex in the inferior frontal gyrus (pars triangularis and pars opercularis); and the motor hand region in the precentral gyrus. The study included 25 congenitally deaf participants and 41 hearing participants (of which 16 were native sign language users); all were right-handed. Deaf participants exhibited a larger calcarine volume than hearing participants, which we interpret as the likely result of cross-modal compensation and/or dynamic interactions within sensory neural networks. Deaf participants also had increased volumes of the pars triangularis bilaterally compared to hearing signers and non-signers, which we interpret is related to the increased linguistic demands of speech processing and/or text reading for deaf individuals. Finally, although no statistically significant differences were found in the motor hand region for any of the groups, the deaf group was leftward asymmetric, the hearing signers essentially symmetric and the hearing non-signers were rightward asymmetric – results we interpret as the possible result of activity-dependent change due to life-long signing. The brain differences we observed in visual, motor, and language-related areas in adult deaf native signers provide evidence for the plasticity available for cognitive adaptation to varied environments during development.

## INTRODUCTION

Across the lifespan, the structural and functional plasticity of the mammalian brain is expressed through a variety of mechanisms and pathways. Several factors influence the potential plastic response of the brain to environmental influences. The extreme and inherent plasticity of the brain in early development is “braked” by a variety of cellular processes that serve ultimately to demarcate a critical or sensitive learning period, during which highly stable functional pathways are trained and established (Bavelier et al., 2010). Cortical plasticity is expressed in numerous brain regions, including both motor and sensory pathways (Dunlop, 2008; Fu and Zuo, 2011). Brain changes associated with various forms of skill learning have been investigated to address questions of brain plasticity. For example, multiple changes in brain structure and function have been detected in musicians compared to non-musicians (Gaser and Schlaug, 2003; Wan and Schlaug, 2010). Another way brain plasticity has been investigated is by studying individuals whose sensory experiences or cognitive demands, often beginning in childhood, are profoundly different from normal experience. For example, blind individuals show changes in brain anatomy that correlate with the length of time blindness has been experienced, with early-onset blind individuals showing more pronounced changes than late-onset individuals (Noppeney, 2007; Leporé et al., 2010a).

A potentially valuable source of insights into the structural and functional plasticity of the human brain is the neurocognitive study of deaf and hearing individuals who are native users of a sign language (MacSweeney et al., 2008; Emmorey and McCullough, 2009). Congenitally deaf signers are particularly interesting for the study of brain plasticity because changes in their structural neuroanatomy could reflect the effects of both skill learning (i.e., the acquisition of a signed language) and sensory deprivation. Given the intense long-term visual and manual experiences associated with learning a sign language from birth, both deaf and hearing signers may be expected to provide evidence of plasticity associated with learning a demanding cognitive and motor skill. Indeed, this prediction has been supported by functional neuroimaging studies. Sign language production activates many of the same areas of the brain as spoken language, but the additional activation of the left parietal lobe in sign language production provides evidence of the brain’s capacity for functional plasticity in response to novel demands (Emmorey et al., 2007; MacSweeney et al., 2008). Structural differences in the insula have also been documented in both hearing and deaf signers, compared to non-signers and have been interpreted as a result of sign language expertise (Allen et al., 2008a).

Due to auditory deprivation during critical developmental periods, some neural changes should be evident in deaf compared to hearing individuals. Auditory regions of the brain are an obvious candidate for plastic changes, and we have shown that deaf people have decreased white matter in and around Heschl’s gyrus, the anatomical landmark for the primary auditory cortex (Emmorey et al., 2003); this finding was later replicated by Shibata (2007). This difference presumably arises as a result of decreased levels of connectivity within, into, and out of the primary auditory cortex, even if this region is co-opted for other functions in deaf individuals (Bavelier and Hirshorn, 2010). We have also shown that deaf individuals have an increased volume in the cortex of the posterior insula, which is not present in hearing signers (Allen et al., 2008a). The increased volume of the posterior insula may be related to the dependence of deaf individuals on lip reading for speech comprehension; functional neuroimaging has shown that deaf, but not hearing, individuals activate the insula during speech reading (MacSweeney et al., 2001).

Here we investigate the effects of sensory deprivation and sign language expertise on plasticity in the human brain by comparing regional cortical volumes in adult, right-handed congenitally deaf signers, hearing signers who acquired American Sign Language (ASL) from birth from their deaf families, and hearing non-signers. We used high-resolution magnetic resonance imaging (MRI) and anatomical landmark-based morphometry to directly measure the cortical volumes of three important and informative regions incorporating visual, language, and motor areas: (1) a primary sensory cortical region – the cortex surrounding the calcarine sulcus in the medial occipital lobe, a proxy for the primary visual cortex or Brodmann area (BA) 17 (Amunts et al., 2000); (2) an association cortex region important for language – Broca’s area within the left inferior frontal gyrus (IFG), and its right hemisphere homolog, subdivided into pars opercularis and pars triangularis (Keller et al., 2007); and (3) a primary motor region – the motor-hand cortex, or handknob, located within the precentral gyrus (Yousry et al., 1997). For each of these three regions, automated volumetry/densitometry research on deaf individuals has indicated that structural differences may arise as a result of auditory deprivation or sign language experience (Penhune et al., 2003; Leporé et al., 2010b; Pénicaud et al., 2012). We look to confirm and expand upon these results using a region of interest (ROI) approach with manually delineated regions and volume calculations performed on individual subjects.

Based on previous studies of enhanced visual ability in deaf individuals (for recent reviews, see Bavelier et al., 2006; Pavani and Bottari, 2012), we predicted that the calcarine cortex volume should show a difference between the deaf group and the two hearing groups in accordance with such altered visual ability. Bottari et al. (2010, 2011) have recently shown that congenitally deaf adults exhibit enhanced “visual reactivity” (superior performance on speeded visual detection tasks), which was associated with very early event-related potential (ERP) changes in striate cortex. These neurobehavioral changes might also co-occur with macroanatomical changes in striate cortex for the deaf group. Difference in gray matter volume for deaf native signers might also be linked to the early age at which sign language acquisition took place; Pénicaud et al. (2012) recently reported that deaf late learners of a sign language exhibited reduced gray matter density in primary visual cortex.

Previous studies also suggest that frontal language regions may be modified by sign language experience or deafness. In a tensor-based morphometry (TBM) study with 14 deaf native signers, Leporé et al. (2010b) found that white matter volumes in Broca’s area and the right frontal operculum were significantly larger for the deaf group compared to hearing non-signing controls. Functionally, Broca’s area and its right homolog have been shown to be involved in sign language comprehension (e.g., Neville et al., 1998; MacSweeney et al., 2002). In addition, functional neuroimaging studies that examine phonological processing of speech and reading in deaf signers report greater activation in Broca’s area and its right hemisphere homolog compared to hearing non-signers (MacSweeney et al., 2009; Corina et al., 2012). Thus, it is currently unclear whether sign language experience or deafness (and the consequent changes in spoken language processing) influences the size of inferior frontal language-related cortices. The inclusion of hearing native signers in our study will help to tease apart these two possibilities.

Finally, Penhune et al. (2003) conducted a voxel-based morphometry (VBM) study with a small group of deaf native signers (*N* = 12) and reported increased gray matter density in the left motor hand area compared to hearing non-signers. The authors hypothesized that increased fine motor control of the dominant hand during signing contributed to volume increases in this area. If this hypothesis is correct, then we should observe differences in the size and/or asymmetry of the motor cortex in the hand region (the handknob) for the signing participants (both deaf and hearing) compared to the non-signers. However, changes in motor cortex may be even more pronounced for deaf signers because ASL is their dominant and most frequently used language compared to hearing signers (see Emmorey et al., 2013a).

In sum, we directly measured the cortical volumes of primary visual, language-related, and motor regions that may be affected by the early acquisition and life-long use of a signed language and/or by congenital deafness. The inclusion of a group of hearing native signers allows us to tease apart effects of deafness from sign language-related effects.

## MATERIALS AND METHODS

### PARTICIPANTS

Participants were 25 congenitally deaf individuals who were native ASL signers (14 women and 11 men, average age = 23.8 years, SD = 4.1, range = 19–38), 25 hearing individuals with no knowledge of ASL (14 women and 11 men, average age = 28.5 years, SD = 4.5, range = 22–39), and 16 hearing individuals who acquired ASL as young children from their deaf parents (10 women and 6 men, average age = 24.3 years, SD = 4.4, range = 19–38). The hearing signers (who had no history of hearing loss) were born into deaf signing families and acquired expertise in ASL as children, concurrently with their acquisition of spoken language. The hearing non-signers were monolingual English speakers with no history of hearing loss. All subjects were right-handed, with scores on the Oldfield–Geschwind Handedness Inventory > +90 (maximum right-handed score +100). All subjects were healthy with no history of neurological or psychiatric illness. Twenty-one deaf subjects exhibited profound hearing loss (>90 dB in the better ear), three subjects had severe hearing loss (>75 dB in the better ear), and one subject had moderately severe hearing loss (>55 dB in the better ear). Data on early hearing aid use was available for 17 deaf subjects. None used a hearing aid consistently before the age of 2. Eleven were required to wear hearing aids at school, but only two also wore hearing aids at home, and six subjects did not wear hearing aids at home or at school. All deaf subjects were congenitally deaf and were born to deaf parents, and ASL was their primary and first language. All subjects gave informed consent in accordance with institutional and federal rules.

### MR IMAGE ACQUISITION AND PROCESSING

Brain image data were collected as previously described (Emmorey et al., 2003; Allen et al., 2008a). Thin cut MR images were obtained in a GE Signa scanner operating at 1.5 T by using the following protocol: SPGR/50, TR 24, TE 7, NEX 1 matrix 256 × 192, field of view (FOV) 24 cm. We obtained 124 contiguous coronal slices, 1.5 or 1.6 mm thick and interpixel distance 0.94 mm. Three individual 1NEX SPGR datasets were obtained for each subject during each imaging session. These were coregistered and averaged *post hoc* using Automated Image Registration (AIR 3.03, UCLA; Woods et al., 1992; Holmes et al., 1998).

Magnetic resonance image analysis (3D reconstructions and volume determinations from ROIs) were conducted using Brainvox (Frank et al., 1997), an interactive family of programs designed to reconstruct, segment, and measure brains from MR acquired images. An automated program, extensively validated against human experts, was used to segment the images into the three primary tissue types (white, gray, cerebral spinal fluid; Grabowski et al., 2000). Although the ROIs may include white matter in the tracings, only gray matter (cortical) volumes are reported. Before tracing ROIs, brains were realigned (but *not* resized; see Allen et al., 2008b for discussion) along a plane running through the anterior and posterior commissures (i.e., the AC–PC line); this procedure ensured that coronal slices in all subjects were perpendicular to a uniformly and anatomically defined axis of the brain.

### REGIONS OF INTEREST

Regions of interests were traced by hand in each hemisphere (by John S. Allen except for the handknob, see below) on contiguous coronal or axial slices of the brain. The study focused on three cortical ROIs: the cortex surrounding the calcarine sulcus (corresponding to BA17), the pars triangularis and opercularis of the IFG (corresponding to BA45 and BA44, respectively and known as Broca’s area in the left hemisphere), and the primary hand motor cortex (corresponding to a sector of BA4) and clearly visible on axial and parasagittal MRI slices (Yousry et al., 1997). Total cerebral hemisphere gray matter volumes (excluding the basal ganglia) were determined as described in Allen et al. (2003).

**FIGURE 1:**
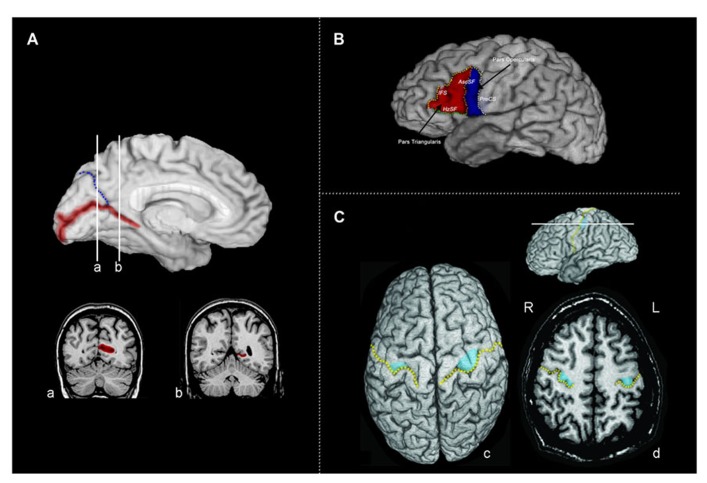
**(A)** The calcarine sulcus. The calcarine sulcus cortex (red) ROI projected to the mesial surface of the left hemisphere. Following Brodmann’s demarcation of area 17, both banks were traced posterior to the intersection of the calcarine sulcus and the parieto-occipital sulcus (blue) (coronal slice **a**), but only the lower bank of sulcus was traced anterior to this intersection (coronal slice **b**). **(B)** Broca’s area (left hemisphere) of the inferior frontal gyrus divided into the pars triangularis (PTri, red) and pars opercularis (POp, blue). Regions of interest were defined and traced as described in Section “Materials and Methods,” following Keller et al. (2007). IFS, inferior frontal sulcus (yellow); AscSF, ascending branch of the Sylvian fissure (coral); HzSF, horizontal branch of the Sylvian fissure (green); PreCS, precentral sulcus (white). **(C)** Tracing the motor handknob. **(c)** The position of the motor handknob (blue) is projected to the surface of the precentral gyrus. The central sulcus is indicated by the broken gold line. **(d)** An axial slice demonstrating the two classic forms of the handknob: the epsilon form in the right hemisphere and the omega form in the left hemisphere. All volumetric tracing of the handknob was done on contiguous 1-mm axial slices. R, right; L, left.

Following Brodmann’s demarcation of area 17 (primary visual cortex or striate cortex), both banks of the calcarine sulcus were traced posterior to the intersection with the parieto-occipital sulcus; anterior to this point, only the lower bank was traced (**Figure 1A**). The ROI of the calcarine cortex included all side branches until its termination at the occipital pole. The surface cortex included was determined by extending a line from the cortical depth of the banks of the sulcus proper to the pial surface. It is important to note that while the calcarine sulcus is a strong anatomical landmark indicating the position of the primary visual cortex, the striate cortex itself extends variably onto the mesial surface of the occipital lobe surrounding the calcarine sulcus (Amunts et al., 2000; Hinds et al., 2008, 2009). The proportion of striate cortex found within the calcarine may only be on the order of 60% (Stensaas et al., 1974; Rademacher et al., 1993); however, both the depth of the calcarine sulcus and the surface area of calcarine cortex correlate to overall striate cortex volume (Gilissen and Zilles, 1996). Thus, the volume of the calcarine cortex is also likely to be correlated with the overall volume of the primary visual cortex.

Tracing of Broca’s area and the homologous region in the right hemisphere (consisting of the pars triangularis and pars opercularis) was done following Keller et al. (2007). As illustrated in **Figure 1B**, the boundaries for the pars opercularis were (1) anteriorly, the ascending branch of the Sylvian fissure (AscSF); (2) posteriorly, the inferior precentral sulcus (PreCS); (3) superiorly, the inferior frontal sulcus (IFS); and (4) inferiorly, the Sylvian fissure (SF). The ascending branch of the SF does not typically reach the IFS, therefore a line connecting the superior end of the ascending branch of the SF to the IFS was drawn perpendicular to the AC–PC plane to close this boundary. When the IFS did not reach the inferior PreCS, a direct line, parallel to the AC–PC plane, from the posterior end of the IFS was used to close the superior boundary. The boundaries for the pars triangularis were: (1) posteriorly, the anterior boundary of pars opercularis formed predominantly by the ascending branch of the SF; (2) inferiorly, the horizontal branch of the SF (HzSF); and (3) antero-superiorly, the IFS, as it extends anteriorly and inferiorly toward the anterior end of the HzSF. A line was drawn from this point to the antero-inferior end of the IFS to close off the anterior boundary. If the IFS was broken into two or more segments, connecting lines were drawn to link the segments. The pars triangularis and opercularis ROIs included all of the cortex located within the boundaries. Gray matter volumes for Broca’s area and its right hemisphere homolog were determined by summing the volumes of the pars opercularis and triangularis.

The primary hand motor region is located in the superior portion of the precentral gyrus of the frontal lobe (**Figure 1C**). It can be identified anatomically in MRI by a sector of the precentral gyrus projecting into the central sulcus (the handknob), which is clearly visible in the axial plane; its position can also be seen in both parasagittal and coronal slices (Yousry et al., 1997; Sastre-Janer et al., 1998; Boling et al., 1999). In the axial plane, the handknob has characteristic omega (single gyrus) and epsilon (double gyrus) forms. The handknob was traced on contiguous axial cuts (1.0 mm thick), which were parallel to the AC–PC plane; its identity and location were confirmed by observation of its hook-like appearance in the sagittal plane. Tracing proceeded superiorly and inferiorly from the axial plane in which the omega or epsilon shaped handknob was identified. The boundaries for the handknob were the central sulcus and a line linking the depths of its limiting lateral and medial sulci. The handknob was traced independently by two investigators (John S. Allen and Joel Bruss), and then a consensus tracing was chosen following review of the tracings by both investigators. Tracing of the handknob was done blind to participant group membership. In addition to the handknob, the entire precentral gyrus was also traced. On the lateral surface, its limits were the PreCS, central sulcus, and the SF. On the mesial surface, the paracentral sulcus was followed as an extension of the superio-mesial portion of the PreCS, and a line was drawn (perpendicular to the AC–PC plane) from the end of the paracentral sulcus to the cingulate sulcus to form the mesial anterior boundary. The posterior mesial boundary was formed by the ascending branch of the cingulate sulcus; a line linking the end of this sulcus to the end of the central sulcus on the mesial surface completed this boundary.

### DATA ANALYSIS

Statistical analyzes were performed using SPSS 19 for Windows (IBM SPSS, Armonk, NY, USA). For each ROI, main effects of hemisphere (left, right) and group (deaf, hearing non-signers, hearing signers) were assessed using a 2 × 3 repeated measures analysis of variance (ANOVA) with total cerebral cortical (gray matter) volume as the covariable. *Post hoc* univariate ANOVAs with total cerebral cortical volume as covariable were used to compare group means (deaf vs. hearing non-signers, deaf vs. hearing signers, hearing signers vs. hearing non-signers). Hemispheric asymmetries were also examined with a conventional asymmetry index (AI) {(*L* - *R*)/[(*L* + *R*)/2]}.

## RESULTS

Volumes of major brain regions are often correlated for size (Allen et al., 2002), therefore before undertaking group-wise comparisons of regional brain volumes, it is important to examine if overall brain size is an important covariable. Cerebral gray matter volume was not significantly different among the three subject groups (*F* = 1.136; df = 2,63; *p* = 0.328); however, the average volume of the hearing signer group was somewhat smaller (587cc, SD = 58.3) than the deaf group (618cc, SD = 56.8) and the hearing non-signer group (606cc, SD = 74.0). Therefore, group differences for each of the target regions were assessed with total cortical hemisphere volume as a covariable.

### CALCARINE SULCUS CORTEX VOLUME

**FIGURE 2:**
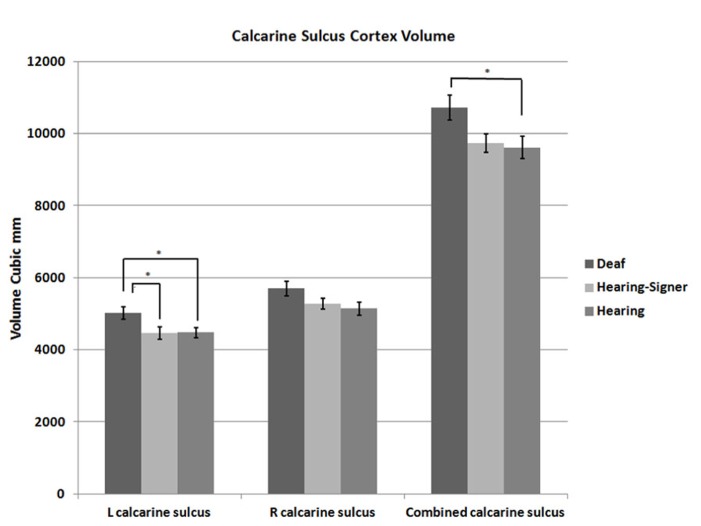
**Calcarine sulcus cortex volume.** Deaf individuals have significantly larger calcarine volume than hearing individuals (error bars: ±1 standard error; *p < 0.05).

The mean volumes of the left and right calcarine sulcus cortex are presented in **Table 1** and **Figure 2**. The 2 × 3 repeated measures ANOVA showed a significant main effect for hemisphere (*F* = 7.295; df = 1,62; *p* = 0.009). The volume of the calcarine cortex was strongly rightwardly asymmetric in all three groups. AI scores were -0.124 (SD = 0.120) for the hearing non-signers, -0.108 (SD = 0.166) for the deaf signers, and -0.161 (SD = 0.156) for the hearing signers. There were no significant differences among the groups for AI score. Within each group, pair-sampled *t*-tests showed that the right calcarine was significantly larger than the left (all *p*s ≤ 0.001). The interaction between hemisphere and participant group was not significant.

**Table 1:** Cortical volumes of the left, right, and total (L + R) calcarine cortex.

	Deaf signers	Hearing signers	Hearing non-signers
L calcarine cortex	5022 (838)	4464 (697)	4475 (733)
R calcarine cortex	5701 (1013)	5276 (604)	5139 (947)
L + R calcarine cortex	10723 (1679)	9740 (1027)	9615 (1570)

*Volumes in mm^*3*^; L, left; R, right; standard deviations are in parentheses.*

The 2 × 3 ANOVA also showed a significant effect of group (*F* = 3.241; df = 2,62; *p* = 0.046). *Post hoc* group-wise comparisons showed that total calcarine volume was significantly larger in the deaf participants compared to hearing non-signing participants (*F* = 5.251; df = 1,47; *p* = 0.026). There were no significant differences between the hearing signing and non-signing participants. Examining each hemisphere separately, we found that the deaf participants’ left calcarine volume was significantly larger than for both the hearing non-signers (*F* = 5.412; df = 1,47; *p* = 0.024) and the hearing signers (*F* = 4.295; df = 1,38; *p* = 0.045). The right calcarine was also larger in the deaf group compared to the other two groups, but the differences were not significant.

### PARS TRIANGULARIS AND OPERCULARIS

**FIGURE 3:**
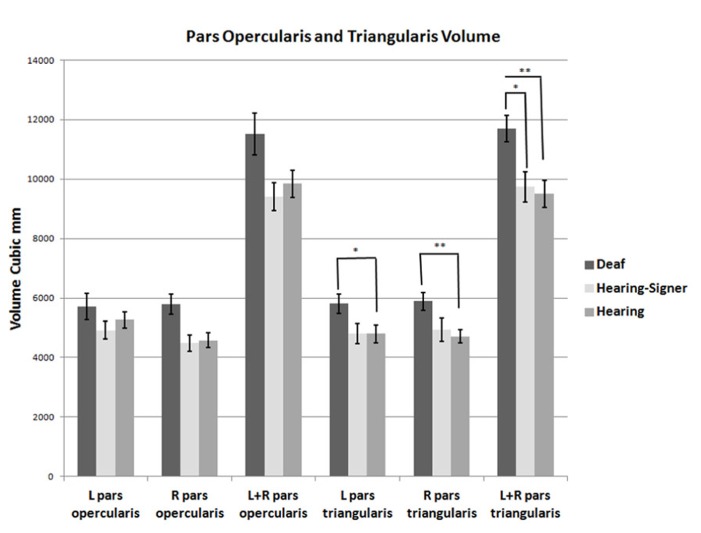
**Volumes of the pars opercularis and pars triangularis.** The pars triangularis is significantly larger in deaf compared to hearing individuals (error bars: ±1 standard error; *p < 0.05; **p > 0.01).

The volumetric results for the pars triangularis and pars opercularis for the left and right hemispheres are presented in **Figure 3** and in **Tables 2** and **3**. For the pars opercularis, there were no main effects for hemisphere or group, although the latter approached statistical significance (*F* = 2.672; df = 2,62; *p* = 0.077). There was no interaction between hemisphere and participant group.

**Table 2:** Cortical volumes of pars opercularis, pars triangularis, and combined pars triangularis and opercularis.

	Deaf signers	Hearing signers	Hearing non-signers
L pars opercularis	5716 (2152)	4914 (1204)	5262 (1406)
R pars opercularis	5793 (1746)	4484 (1099)	4582 (1210)
L + R pars opercularis	11509 (3518)	9398 (469)	9844 (2258)
L pars triangularis	5811 (1590)	4809 (1330)	4801 (1514)
R pars triangularis	5886 (1463)	4928 (1552)	4705 (1099)
L+R pars triangularis	11698 (2271)	9736 (2015)	9506 (2252)

ANCOVA results for the effect of group (using hemisphere cortical volume as a covariable). Bonferroni-corrected *t*-tests were used for *post hoc* group-wise comparisons. Volumes in mm^*3*^; L, left; R, right; standard deviations are in parentheses, *P ≤ 0.05; **P ≤ 0.01.

**Table 3:** Asymmetry index scores for the pars opercularis and pars triangularis.

	Deaf signers	Hearing signers	Hearing non-signers
Pars opercularis asymmetry index	-0.0306 (0.2725)	0.0912 (0.2911)	0.1332 (0.2776)
Pars triangularis asymmetry index	-0.0184 (0.3211)	-0.0142 (0.4280)	-0.0006 (0.2974)

*Asymmetry index: {(*L* - *R*)/[(*L* + *R*)/2]}.*

For the pars triangularis, there was no significant main effect of hemisphere or interaction between participant group and hemisphere. However, there was a significant main effect of group (*F* = 6.627; df = 2,62; *p* = 0.002). *Post hoc* tests showed the pars triangularis volumes were significantly larger for the deaf signers compared to the hearing non-signers for the combined volume (L + R; *F* = 11.662; df = 1,47; *p* = 0.001), for the left hemisphere volume (*F* = 4.824; df = 1,47; *p* = 0.033), and for the right hemisphere volume (*F* = 9.678; df = 1,47; *p* = 0.003). A similar pattern was seen comparing the deaf signers to the hearing signers, with the combined volume (L + R) difference reaching statistical significance (*F* = 4.889; df = 1,38; *p* = 0.033). There were no significant differences between the hearing non-signers and hearing signers in the pars triangularis volumes.

### MOTOR HANDKNOB CORTEX

**FIGURE 4:**
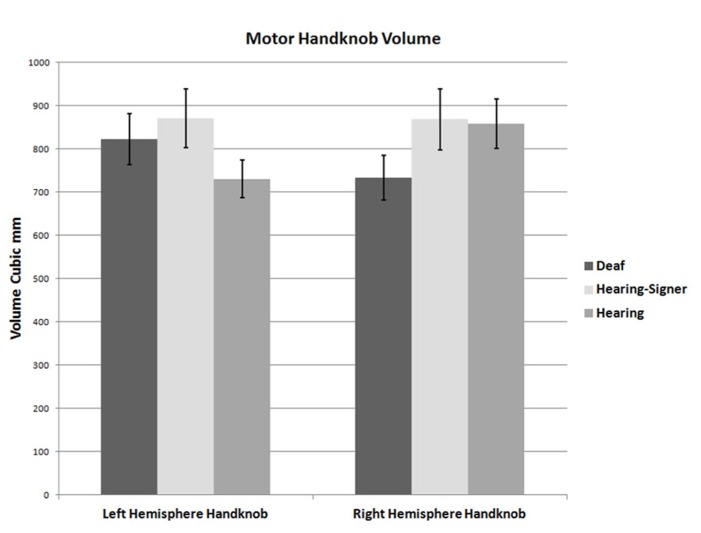
**Volumes of the left and right handknobs (error bars: ±1 standard error)**.

Motor handknob cortical volume results by hemisphere are presented in **Figure 4** and **Table 4**. The 2 × 3 ANOVA showed no significant main effects of hemisphere or group, and no interaction. It is interesting to note, however, that the asymmetry indices of the three groups did vary substantially. The deaf individuals were leftwardly asymmetric, with the left hemisphere handknob being about 11% larger than the right, while the hearing non-signers were rightwardly asymmetric to an even greater degree (about 14% larger on the right). The hearing signers had essentially symmetrical handknobs. Comparing the deaf signers to the hearing non-signers (leaving the hearing signers out of the model), the AI score was significantly different between the two groups (*F* = 5.243; df = 1,47; *p* = 0.027).

**Table 4:** Motor handknob cortical volumes and asymmetry indexes.

	Deaf signers	Hearing signers	Hearing non-signers
Left hemisphere handknob	822 (296)	871 (270)	730 (216)
Right hemisphere handknob	733 (260)	868 (284)	858 (288)
Handknob asymmetry index	0.115 (0.401)	0.014 (0.393)	-0.139 (0.416)

*Asymmetry index: {(*L* - *R*)/[(*L* + *R*)/2]}.*

*All volumes in mm^*3*^; standard deviations are in parentheses.*

## DISCUSSION

The results presented here expand our perspective on the scope of structural cortical plasticity associated with congenital deafness and life-long signing. We found significant differences between the deaf and hearing groups in the cortex of the calcarine sulcus and the pars triangularis, while the handknob results suggested that this region may be lateralized differently in deaf signers compared to hearing non-signers. In general, the hearing signers were more similar to the hearing non-signers than to the deaf signers, suggesting a primary influence of auditory deprivation in shaping neuroanatomical plasticity. This is not to say that the differences observed between deaf and hearing individuals were solely due to lack of auditory experience, but that the combination of sign language usage and deafness may have had a large role in shaping the cortical differences we observed.

### THE CALCARINE SULCUS: SENSORY COMPENSATION AND MULTISENSORY INTEGRATION

There has been longstanding interest in the question of whether or not compensatory visual enhancement accompanies congenital deafness (Bavelier et al., 2006). We examined the cortex surrounding the calcarine sulcus (as a proxy for BA17, the primary visual cortex) to determine whether there was evidence of anatomical changes in deaf compared to hearing individuals. We had two major findings concerning the volume of the cortex surrounding the calcarine sulcus. First, for all three participant groups, the right calcarine cortex volume was larger than the left; this pattern was indicated both by AI scores and pair-wise *t*-tests. A rightward gray matter asymmetry in the cortex of the medial occipital lobe in the neighborhood of the calcarine sulcus has been observed in several studies using automated assessment methods (VBM or cortical thickness; Good et al., 2001; Hervé et al., 2006; Luders et al., 2006; Takao et al., 2011; although see Watkins et al., 2001). Our results confirm these findings and provide a volumetric perspective on this asymmetry in native-space (i.e., non-transformed) brain images. Consistent with our results and some of the VBM studies, a direct histological volumetric assessment of the striate cortex has also shown that the right side is significantly larger than the left (Murphy, 1985). A rightward asymmetry of the striate cortex is consistent with the notion that visuo-spatial function is more related to right hemisphere function (Corballis, 2003).

We also found evidence of increased total volume of the calcarine cortex in the deaf group compared to the two hearing groups (see **Figure 2**). Given the positive correlation between intracalcarine striate cortex volume and total striate cortex volume (Gilissen and Zilles, 1996), our findings suggest that deaf individuals on average may have more primary visual cortex than hearing individuals (although such a conclusion would require histological verification). One study using VBM has shown increased size in the visual cortex of deaf compared to hearing subjects, especially on the right side (Leporé et al., 2010b). The increase was interpreted as evidence of possible sensory compensation for the absence of auditory input. In contrast, Pénicaud et al. (2012) found reduced gray matter density in visual cortex for deaf individuals, but only for those who learned sign language as a first language late in childhood. Pénicaud et al. (2012, p. 7) reported increased density for deaf native signers (compared to hearing non-signers), and they hypothesized that early ASL acquisition is associated with the increase in gray matter density, which may arise because of “greater computational power in the visual cortex leading to a more efficient visual-perceptual analysis of the sign language signal.” However, given that native hearing signers in our study did not differ significantly from hearing non-signers, our findings suggest that it may be the *combination* of auditory deprivation and exposure to ASL in infancy that leads to a significant increase in gray matter volume within the calcarine cortex.

If increased size of the visual cortex in deaf individuals is at least in part a result of visual compensation for deafness, there could be evidence of altered visual abilities in deaf compared to hearing individuals. Although a fair amount of research has addressed these questions, deaf individuals do not seem to perform all that differently from hearing individuals on a range of visual-perceptual threshold tasks, such as coherent motion or flicker-fusion thresholds (for reviews, see Bavelier et al., 2006; Pavani and Bottari, 2012). However, several studies have shown that deaf individuals are significantly faster to detect abrupt visual onsets (e.g., Loke and Song, 1991; Reynolds, 1993), and Bottari et al. (2011) recently showed that this ability is correlated with ERP components in striate cortex (the C1 component, 80 ms from stimulus onset). The increased cortical volume found in the calcarine sulcus for deaf individuals might be related to this enhanced ability to rapidly react to visual stimuli. In addition, the most robust differences between deaf and hearing people are seen on tests that depend on peripheral vision. Deaf individuals have a significant advantage in attending to peripheral stimuli, which results in a relatively expanded FOV (Dye et al., 2009). One manifestation of this finding is that deaf individuals are more influenced by peripheral distractors than central distractors, the opposite of the pattern observed for hearing individuals (Proksch and Bavelier, 2002). In deaf individuals the increased peripheral sensitivity is correlated with an increase in the area of the neural rim of the retina, suggesting that the advantage is in fact due to early sensory changes (Codina et al., 2011). Thus it is possible that the expanded calcarine cortex in deaf individuals is also related to expanded attention to the periphery of the visual field.

In addition to a compensatory model for the possible expansion of striate cortex in deaf signers, the dynamic nature of neuronal and synaptic plasticity that occurs in the context of auditory and visual convergence, or multisensory activation (Bavelier and Hirshorn, 2010) may also contribute to anatomical changes. Integration between primary visual and auditory cortices is pervasive, at both the structural and functional levels (Kayser and Logothetis, 2007; Raij et al., 2010). Cortical plasticity is thus very much a function of dynamic interactions within neural networks, and the changes in the calcarine that we observed may be a result of both visual compensation for auditory deprivation and changes in multisensory integration networks. Further research is needed to clarify the potential contributions of each of these factors in shaping the structural and functional anatomy of the deaf primary visual cortex.

### PARS TRIANGULARIS AND PARS OPERCULARIS: EFFECTS OF DEAFNESS ON LANGUAGE-RELATED CORTICES

Our results revealed that the pars triangularis (bilaterally and combined) was significantly larger in the deaf group compared to either of the hearing groups. The pars opercularis showed no significant group differences, although there was a trend for larger volumes in the deaf group. These results partially replicate the findings of Leporé et al. (2010b). Given that our finding pertains *only* to the deaf group, it suggests that the increased volume in the pars triangularis is associated with deafness, rather than with life-long signing. The IFG (pars triangularis and opercularis) is known to be involved in a variety of linguistic and cognitive functions that may differ for deaf and hearing individuals (e.g., text reading, speech production and comprehension, working memory, human action recognition, etc.). However, we speculate that the increased demands faced by congenitally deaf individuals for deciphering spoken language through both print and lip reading may underlie the increased cortical volumes observed here.

For deaf children, access to sound-based phonology is greatly reduced or absent, and many struggle to learn to read. Recent neuroimaging studies with deaf adults have reported that when the reading task requires phonological processing, deaf readers exhibit greater activation in the IFG (both pars triangularis and pars opercularis) compared to hearing readers (Aparicio et al., 2007; Emmorey et al., 2013b). Less skilled deaf readers also show greater right inferior frontal activation in an implicit reading task compared to more skilled deaf readers (Corina et al., 2012). Furthermore, MacSweeney et al. (2009) found that adult deaf readers and hearing dyslexic individuals showed increased activation of the left IFG during a phonological rhyming judgment (compared to hearing normal readers). Phonological processing is disrupted in both deaf and dyslexic individuals (for different reasons), and increased activation in left IFG appears to arise as a compensatory mechanism during phonological tasks. Thus, it is possible that the demands of learning to read a language that one cannot hear exacts more intense processing in core association areas, including both the left and right inferior frontal gyri, leading to their increase in size.

In addition, the demands of acquiring spoken language primarily from visual input may also lead to an increase in the volume of the pars triangularis. For deaf individuals, speech reading will have to rely, necessarily, on visual and articulatory rather than auditory cues. The increased volume of Broca’s area (mainly pars triangularis) shown here, and the increased volume of the left posterior insula described earlier (Allen et al., 2008a), may represent evidence for a speech reading network in deaf individuals linking language processing and multisensory integration.

### SIGN LANGUAGE AND MOTOR HANDKNOB ASYMMETRY

The handknob is a reliable landmark for the position of the hand primary motor cortex in the precentral gyrus (M1; Yousry et al., 1997; Sastre-Janer et al., 1998). However, the anatomy of the handknob displays considerable inter-individual variation, which may make it difficult to detect structuro-functional changes that might arise due to activity or experience (Caulo et al., 2007; Boling et al., 2008).

Surprisingly, an expected direct link between anatomy (the volume or gray matter density of the handknob) and handedness has not been definitively established. The strong expectation is that the handknob would be larger on the contralateral side to the dominant hand. Direct evidence to support or refute this expectation has been lacking, possibly because there have been very few direct volumetric studies of the human handknob, and various proxy or indirect measurements have produced equivocal results (see Amunts et al., 1996 vs. Davatzikos and Bryan, 2002). An automated analysis of global patterns of cortical thickness in the cerebrum shows a leftward asymmetry in the superior portion of the precentral gyrus, a region that would include the handknob (Luders et al., 2006). In contrast, a VBM study using a ROI approach, showed greater gray matter density in a region around the central sulcus near the location of the handknob in the right hemisphere of both left- and right-handers (Hervé et al., 2005). Histological studies have shown that BA 4 volume (the whole of the primary motor cortex including the hand region) is essentially symmetrical in right-handers, with no laterality evident in the region corresponding to the handknob (White et al., 1997; Rademacher et al., 2001). Of course, these studies are measuring different aspects of the motor region, and doing so using quite different methods, although they all exhibit a potential relation to the motor hand cortex.

Our results for right-handed non-signers, based on direct anatomical measurement of the volume of gray matter encompassed by the region corresponding to the hand, are consistent with those studies finding a rightward asymmetry in the handknob. Our AI indicated that the right hemisphere handknob was about 14% larger than the left, a magnitude of asymmetry that is generally considered to be noteworthy. It is interesting to note that neither of the two signing groups exhibited such a rightward asymmetry: the hearing signers were essentially symmetrical, while the deaf signers were actually 11% larger in the left hemisphere. The AI of the deaf individuals was significantly different from that of the hearing non-signers. This intriguing finding together with the finding of essential symmetry in the hearing signers suggests the possibility of activity-dependent plasticity resulting from sign language usage and deserves further investigation.

Our results are also consistent with Penhune et al.’s (2003) hypothesis that the fine motor control of the hands required for signing leads to structural changes in the hand motor region. Furthermore, plastic gray matter changes in the hand region with motor training have been reported (Granert et al., 2011). There are also reports of relative volumetric preservation of the volume of the cortex in the hand regions in aging (both motor and sensory) in the hemisphere contralateral to the dominant hand (Bonilha et al., 2009). These findings support the possibility that the volume of the cortex of the hand region may be maintained by activity. The possible increase of the left handknob cortex in native signers, the language dominant hemisphere, may be indicative not just of differences in motor activity but of the recruitment of this region into a primary language network when signing begins during an early critical period of development.

## CONCLUSION

Congenitally deaf individuals who are native users of sign language showed evidence of structural cortical changes in each of the three brain regions examined. Increased bilateral volumes of the calcarine cortex and the pars triangularis in deaf individuals relative to the volumes of both signing and non-signing hearing individuals provide evidence of likely developmental cortical plasticity in response to an environment of auditory deprivation. Differences in lateralization of the motor hand region observed for deaf and hearing signers may be related to early and prolonged use of sign language. Because of the variability seen in this region, this finding should be further pursued in larger samples. Overall, the study of structural and functional brain anatomy in congenitally deaf signers provides an insight into the variety of forces that influence cognitive and structural adaptation to specific environments.

## Conflict of Interest Statement

The authors declare that the research was conducted in the absence of any commercial or financial relationships that could be construed as a potential conflict of interest.
